# Vascular plant colonisation of Surtsey Island (1965-1990) - a dataset

**DOI:** 10.3897/BDJ.8.e54812

**Published:** 2020-07-07

**Authors:** Pawel Wasowicz, Sally Thorsteinsson, Borgþór Magnússon, Eyþór Einarsson, Valgeir Bjarnason, Ágúst H. Bjarnason, Jón Guðmundsson, Sigurður H Richter, Ragnar Jónasson, Bjartmar Sveinbjörnsson, Skúli Þ Magnússon

**Affiliations:** 1 Icelandic Institute of Natural History, Akureyri, Iceland Icelandic Institute of Natural History Akureyri Iceland; 2 Icelandic Institute of Natural History, Garðabær, Iceland Icelandic Institute of Natural History Garðabær Iceland; 3 Surtsey Research Society, Reykjavík, Iceland Surtsey Research Society Reykjavík Iceland

**Keywords:** Surtsey, Iceland, primary succession, Northern Atlantic, volcanism, colonisation

## Abstract

**Background:**

The process of ecosystem development over time that takes place on a new substrate devoid of biological activity (such as, for example, lava) is called primary succession. Research on primary succession is not easy, as it is limited to rare occasions when a piece of land totally lacking in any pre-existing life occurs. The emergence of volcanic islands is such an occasion; it is a unique event that allows a natural experiment in the study of colonisation processes and primary succession. Surtsey (located in the Vestmannaeyar archipelago off the southern coast of Iceland) is an iconic example of a place where primary succession has been studied for decades and where human disturbance has been minimised due to significant geographic isolation and early protection efforts. Here, we present a georeferenced dataset of vacular plant occurrences collected during the field studies carried out on Surtsey Island during the first three decades of its existence.

**New information:**

To date, no dataset containing plant distribution data documenting the process of early stages of colonisation of Surtsey has been published. What is more, to our knowledge, there is no other dataset that can be compared with our Surtsey data that is readily available for researchers working on plant colonisation dynamics and primary succession processes. Here, we present a complete, geo-referenced dataset of all plant occurrences (10,094 in total) collected on Surtsey between 1965 and 1990.

## Introduction

Surtsey is a volcanic island located approximately 32 km off the south coast of Iceland. It was formed during a volcanic eruption taking place from November 1963 to June 1967 (Þórarinsson 1968). In 1965, the whole island became legally protected due to its special scientific value and the research opportunities that arose with its formation. Indeed, the protection of Surtsey created an isolated island environment, free of human interference, that enabled researchers to collect priceless data documenting the process of colonisation of the newly-formed island.

**Figure 1.**
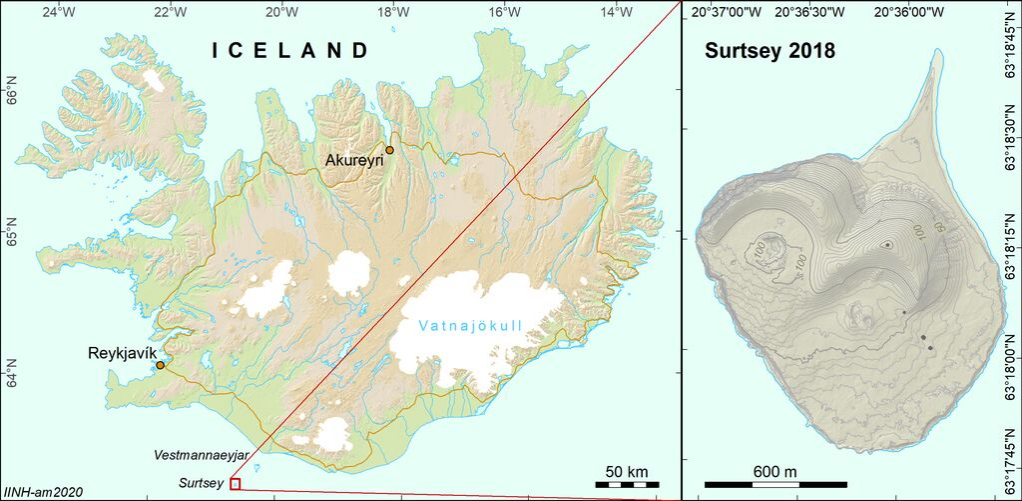
Location and general topography of Surtsey. The coastline of Surtsey as of 2018.

Surtsey is a part of the Vestmannaeyjar archipelago (S Iceland, Fig. 1). The archipelago constitutes a separate volcanic system, consisting of 18 islands and a number of skerries and is situated on the insular shelf off the south coast of Iceland. As a result of volcanic activity between 1963 and 1967, an additional island was formed, Surtsey, that initially had an area of 2.65 km^2^ (Baldursson and Ingadóttir 2007). The total volume of the erupted material was estimated to be about 1.1 km^3^ and consisted of tephra (70%) and lava (30%). The surface of Surtsey has been mapped in detail using traditional methods, but no GIS data are publicly available. The eruptive products at Surtsey, tephra and lava, are composed of alkali basalt with phenocrysts of olivine, plagioclase and chromian spinel. With the passage of time, three secondary geological units were formed: palagonite tuff, aeolian and talus sediments and coastal sediments. The highest elevation on Surtsey is 155 m. It is good to have in mind that the shape of Surtsey is constantly modified by the harsh weather conditions, particularly during winter (Baldursson and Ingadóttir 2007).

Automatic weather measurements in Surtsey have ben carried out since 2009 and were recently summarised by Petersen and Jónsson 2020. The climate here is maritime, with the annual temperature variation dampened by the sea that cools the climate during summer and warms it during winter. Average winter temperatures (December to March) are between 2.5 and 4.0ºC, while average summer temperatures (July and August) are close to 10ºC. The frost-free period lasts here for about 199 days. The highest temperature recorded on Surtsey was 18.3ºC, while the minimum was -9.7ºC. The average annual precipitation is about 1000 mm, with June being the driest month and October being the wettest. Snowfall is rare and rather slight. Winds are strong. On average, there are 229 days per year with precipitation. Average wind speed on Surtsey is 7.8 m/s. There are 30 days (on average) with wind speeds exceeding 20 m/s. The maximum wind speed recorded here was 35.2 m/s, while the maximum wind gust was 47.8 m/s (Petersen and Jónsson 2020).

Botanical observations on Surtsey commenced in May 1964 (Einarsson 1963), when the eruption was still underway and uncovered, even at that time, a significant amount of plant material carried by sea currents that was able to reach the island. A year later (on 3 June 1965), the first vascular plant species (seedlings of Cakile maritima subsp. islandica) was discovered growing on Surtsey (Friðriksson 1966). During 56 years of careful, yearly observations, a significant amount of data on plant distribution and colonisation was collected, resulting in many scientific papers (e.g. Einarsson 1967, Friðriksson 1966, Friðriksson 1978, Friðriksson 1982, Friðriksson 1992, Friðriksson et al. 1972, Friðriksson 1967, Friðriksson and Johnsen 1968, Magnússon et al. 2014). More detailed information on various aspects of research carried out in Surtsey can be found in a series of reports published by Surtsey Research Society. All the reports are available at https://surtsey.is/utgafa-surtseyjarfelagsins/ .

During the first decades of research carried out on Surtsey, every single plant growing on the island was precisely marked (initially using wooden poles), numbered and mapped using coordinate paper. In the present data paper, we publish the results of digitisation of these maps containing observations of plant life during the first decades of the history of Surtsey. This project, recently completed at the Icelandic Institute of Natural History, resulted in a database of 10,094 records documenting the process of colonisation and subsequent spread of vascular plants on Surtsey.

## General description

### Purpose

The present project was focused on digitising the data on plant distribution on Surtsey Island, collected between 1965-1990 by botanists taking part in yearly expeditions to the Island.

## Sampling methods

### Sampling description

**Original data collection**

Original data were collected during field studies carried out on Surtsey between 1964 and 1990. Original maps documenting vascular plant colonisation in 1965 and 1966 were not available. Instead, based on original descriptions and maps of Surtsey from 1965 and 1966, three distribution points were located in squares B12 (Cakile maritima subsp. islandica, the first record from 1965 based on a description from Friðriksson 1966), A12 (Cakile maritima subsp. islandica, record from 1966 based on Friðriksson 1967) and A11 (*Leymus arenarius*, record from 1966 based on Friðriksson 1967). These points were assigned with 300 m coordinate uncertainty in our dataset. It is worth noting that localities of these initial colonisers were short-living and very soon (within weeks) were wiped out by volcanic ash and sea waves.

**Figure 2.**
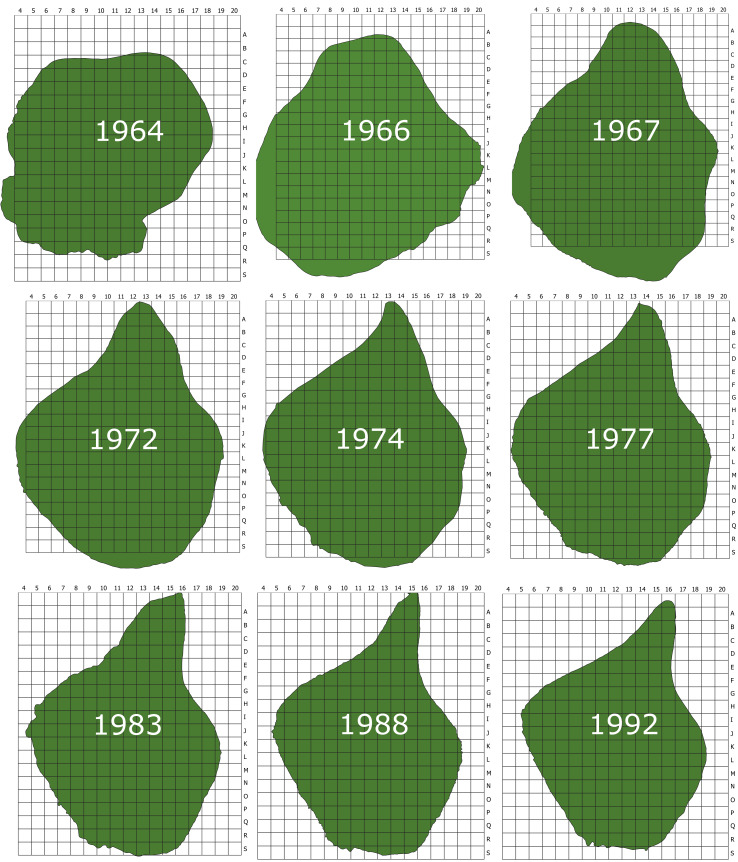
The 1 ha grid used in the mapping of plant distribution on Surtsey and changes of the coastline (caused by coastal erosion) of the island between 1964 and 1992.

**Figure 3.**
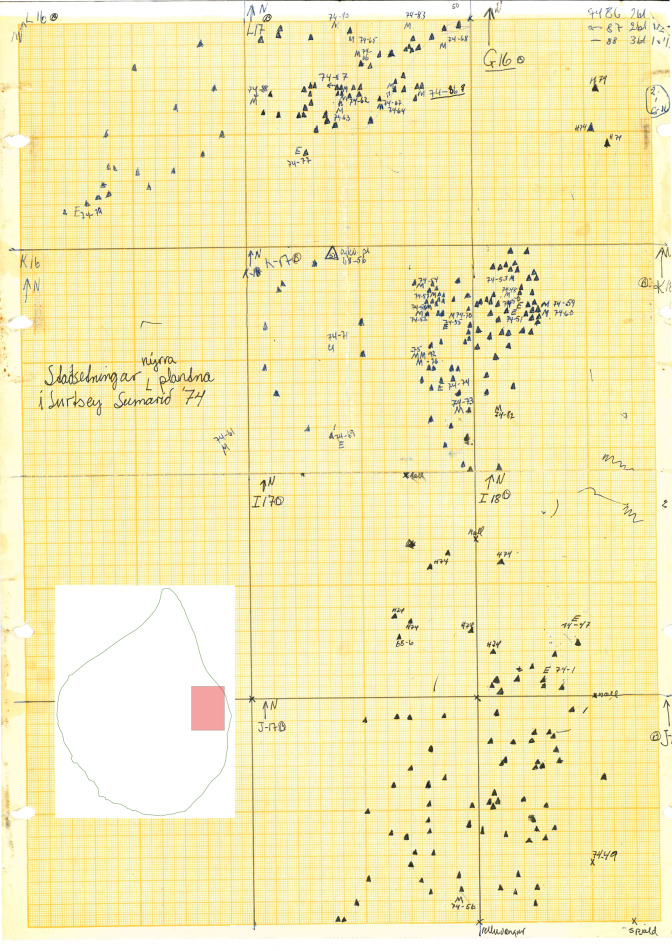
An example of an original map sheet made on coordinate paper and containing the information on the distribution of plant species on Surtsey recorded in 1974. Note that this map is only a fragment covering 1 ha square with numbers L16-18, K16-18, I16-18 and J16-18.

From the year 1967 onwards, the digitisation was based on original distribution maps. The whole area of the Island was divided into 1 ha squares and plant mapping was carried out in each plot (Fig. 2). Between 1964 and 1978, the whole area of the Island was searched annually for the presence of vascular plants. When found, each plant was initially marked with a wooden stick, numbered and subsequently mapped using coordinate paper (an example of such a map can be found in Fig. 3). When the number of plants increased, marking with wooden sticks was discontinued. Mapping activities were also carried out on Surtsey in 1980 (Friðriksson 1982) and in 1990 (Friðriksson 1992).

**Digitisation process and coordinate uncertainty levels**

During the digitisation, each map was scanned and then georeferenced using corners of the net of 1 ha square grid as control points. The number of control points was different for each map, but the minimal number of control points was 10 for a map sheet covering an area of 12 ha (the smallest map digitised). As a rule, each occurrence point on a map corresponds with one point digitised in our dataset. However, when authors of the original map sheets marked an area and indicated the number of individuals growing in the area, the same number of points was distributed randomly within this area during the digitisation process.

From 1980, Honckenya peploides subsp. diffusa became so common on Surtsey that it was impossible to map every single plant and its distribution on original maps was recorded by drawing areas rather than points. We approached this problem in the following way: 1. species distribution was recorded per 1 ha square and distribution points in our dataset represent centroids of each 1 ha plot, where the occurrence of H. peploides subsp. diffusa was confirmed; 2. areas with confirmed presence of the species were digitised as polygons and corresponding shapefiles are available as a separate data resource. This applies only to H. peploides subsp. diffusa in the years 1980 and 1990.

Coordinate uncertainty was experimentally established during the field studies using a set of 30 wooden sticks (used in mapping during the first decade of research). Only sticks with clearly legible numbers were included. Coordinates of these sticks were recorded in the field (using Garmin GPSmap 62s) and then compared with the data obtained from digitisation. The mean uncertainty was established to be 10 m. This level of coordinate uncertainty was used throughout the dataset unless otherwise stated. When points of distribution were recorded as polygon centroids (see above), the uncertainty was set to 50 m. The three initial observations made on Surtsey were assigned 300 m uncertainty level, due to the reasons stated above.

## Geographic coverage

### Description

All the data published in the present paper originate from the island of Surtsey.

### Coordinates

63.288 and 63.315 Latitude; -20.580 and -20.630 Longitude.

## Taxonomic coverage

### Description

The dataset covers 27 vascular plant taxa recorded from Surtsey between 1965 and 1990. Latin nomenclature follows Annotated checklist of vascular plants of Iceland (Wasowicz 2020).

### Taxa included

**Table:** 

Rank	Scientific Name	Common Name
species	*Agrostis stolonifera* L.	Skriðlíngresi
species	*Alchemilla filicaulis* Buser	Maríustakkur
subspecies	Angelica archangelica L. subsp. archangelica	Ætihvönn
species	*Arabiopsis petrea* (L.) V.I. Dorof.	Melablóm
subspecies	Armeria maritima (Miller) Willd. subsp. maritima	Geldingahnappur
species	Cakile maritima subsp. islandica (Gand.) Hyl. ex Elven	Fjörukál
species	*Capsella bursa-pastoris* (L.) Medik.	Hjartarfi
species	*Carex maritima* Gunnerus	Bjúgstör
subspecies	Cerastium fontanum Baumg. subsp. fontanum	Vegarfi
species	*Cochlearia islandica* Pobed.	Skarfakál
species	*Cystopteris fragilis* (L.) Bernh.	Tófugras
species	*Epilobium palustre* L.	Mýradúnurt
subspecies	Equisetum arvense L. subsp. arvense	Klóelfting
species	*Festuca richardsonii* Hook.	Túnvingull
subspecies	Honckenya peploides subsp. diffusa (Hornem.) Hultén ex V.V. Petrovsky	Fjöruarfi
species	*Juncus arcticus* Willd.	Tryppanál
species	*Leymus arenarius* (L.) Hochst.	Melgresi
subspecies	Luzula multiflora subsp. frigida (Buchenau) V.I. Krecz.	Vallhæra
subspecies	Mertensia maritima (L.) Gray subsp. maritima	Blálilja
species	*Poa annua* L.	Varpasveifgras
subspecies	Poa pratensis subsp. irrigata (Lindm.) H. Lindb.	Vallarsveifgras
species	*Puccinellia maritima* (Huds.) Parl.	Sjávarfitjungur
species	*Rumex acetosella* L.	Hundasúra
species	*Sagina procumbens* L.	Skammkrækill
species	*Silene vulgaris* (Moench) Garcke	Garðaholurt
species	*Stellaria media* (L.) Vill.	Haugarfi
subspecies	Tripleurospermum maritimum subsp. subpolare (Pobed.) Hämet-Ahti	Baldursbrá

## Temporal coverage

**Formation period:** 1965-1990.

## Usage rights

### Use license

Other

### IP rights notes

Creative Commons Attribution Non Commercial (CC-BY-NC) 4.0 License

## Data resources

### Data package title

Distribution of vascular plants on the island of Surtsey (Iceland) between 1965 and 1990

### Number of data sets

2

### Data set 1.

#### Data set name

Vascular plants on Surtsey Island (Iceland) point distribution data

#### Number of columns

27

#### Download URL

https://doi.org/10.15468/cht6nd

#### Description

The occurrence of vascular plant species recorded during floristic surveys on Surtsey (Iceland) from 1965 to 1990.

**Data set 1.** 

Column label	Column description
occurrenceID	An identifier for the occurrence (unique).
occurrenceRemarks	Comments or notes about the Occurrence.
recordedBy	A list of names of people, groups or organisations responsible for recording the original Occurrence.
scientificName	The full scientific name, with authorship and date information, if known.
kingdom	The full scientific name of the kingdom in which the taxon is classified.
family	The full scientific name of the family in which the taxon is classified.
genus	The full scientific name of the genus in which the taxon is classified.
specificEpithet	The name of the first or species epithet of the scientificName
infraspecificEpithet	The name of the lowest or terminal infraspecific epithet of the scientificName, excluding any rank designation
taxonRank	The taxonomic rank of the most specific name in the scientificName.
scientificNameAuthorship	The authorship information for the scientificName formatted according to the conventions of the applicable nomenclaturalCode.
vernacularName	A common or vernacular name.
continent	The name of the continent in which the Location occurs.
country	The name of the country or major administrative unit in which the Location occurs.
locality	The specific description of the place.
decimalLatitude	The geographic latitude (in decimal degrees, using the spatial reference system given in geodeticDatum) of the geographic centre of a Location. Positive values are north of the Equator, negative values are south of it. Legal values lie between -90 and 90, inclusive.
decimalLongitude	The geographic longitude (in decimal degrees, using the spatial reference system given in geodeticDatum) of the geographic centre of a Location. Positive values are east of the Greenwich Meridian, negative values are west of it. Legal values lie between -180 and 180, inclusive.
geodeticDatum	The ellipsoid, geodetic datum or spatial reference system (SRS) upon which the geographic coordinates given in decimalLatitude and decimalLongitude as based.
coordinateUncertaintyInMetres	The horizontal distance (in metres) from the given decimalLatitude and decimalLongitude describing the smallest circle containing the whole of the Location. Leave the value empty if the uncertainty is unknown, cannot be estimated or is not applicable (because there are no coordinates).
georeferencedBy	A list (concatenated and separated) of names of people, groups or organisations who determined the georeference (spatial representation) for the Location.
identifiedBy	A list (concatenated and separated) of names of people, groups or organisations who assigned the Taxon to the subject.
dateIdentified	The date on which the subject was identified as representing the Taxon.
year	The four-digit year in which the Event occurred, according to the Common Era Calendar.
month	The ordinal month in which the Event occurred
day	The integer day of the month on which the Event occurred.
basisOfRecord	The specific nature of the data record.
language	A language of the resource.

### Data set 2.

#### Data set name

Distribution of Honckenya peploides ssp. diffusa on Surtsey in 1980 and 1990 (as polygons).

#### Data format

ESRI shape file, CRS: EPSG:3057 - ISN93/Lambert 1993

#### Number of columns

3

#### Download URL

https://doi.org/10.5281/zenodo.3909368

#### Description

The distribution of Honckenya peploides subsp. diffusa in 1980 and 1990 digitised as polygons. The data is available in ESRI shapefile: Honckenya pepl 1980_1990 polygons.shp

**Data set 2.** 

Column label	Column description
ID	An identifier for the occurrence (polygon)
year	The four-digit year in which the event occurred, according to the Common Era Calendar.
species	The full scientific name of the species
